# 

**Published:** 1914-12-12

**Authors:** 


### LEEDS SURGEONS FOR THE FRONT.

The two well-known surgeons—Sir Berkeley Moynihan and Mr. L. R. Braithwaite—left Leeds for the Front last week. Sir Berkeley holds the rank of colonel in the Territorial branch of the Royal Army Medical Corps, and Mr. Braithwaite that of captain. Both gentlemen have gone as consultants in abdominal surgery. Mr. Braithwaite has been closely associated with Sir Berkeley for some years, and for some time he assisted Sir Berkeley at the latter's nursing home.

### Personalia.

A R.A.M.C. PRISONER OF WAR.

Captain A. M. Pollard, R.A.M.C., attached to the 9th Lancers, is a prisoner of war at Gütersloh, Germany, where he is announced to be quite well. He was reported by the War Office as missing on October 31. It is understood that he was captured at Messines on that date while engaged in attending to the wounded.

A VETERAN DOCTOR OF THE COACHING DAYS.

Dr. W. T. Edwards, J.P., of Cardiff, entered upon his ninety-fourth year on Sunday, December 6. He was born at Caerphilly, and started practice in Cardiff when the population was only 12,000. When he left his native town to study at the University College, London, he had to walk as far as Newport, proceed by packet to Bristol, thence to the Metropolis by coach! Dr. Edwards still takes a keen interest in public topics, and tells many a story of the transformation which has come over the face of the country during the last eighty years. He is an M.D. Lond., winning a gold medal at his M.B. examination, and a F.R.C.S. Eng. He has retired from practice.

### The Book Market.

LIST OF NEW BOOKS RECEIVED FROM THE FOLLOWING PUBLISHERS:-

Oxford University Press: "Text-Book of Massage and Remedial Gymnastics." By Ll. Despard. Second Edition. Price 12s. 6d. net.

T. Fisher Unwin: "From the Trenches: Louvain to the Aisne." By Geoffray Young. Price 2s. net. "The Song of the Guns." By Herbert Kaufman. Price 1s. net.

### Medical Appointments.

Mr. Hugh Davies has been appointed a district medical officer under the London County Council, in succession to the late Dr. R. D. Muir.

The London County Council has renewed for a further period of one year the appointment of Mr. Arthur Herbert Spicer as deputy medical examiner to the Council.

Mr. W. W. Coltart, L.R.C.P., M.R.C.S., having resigned the position of medical officer to Epsom College, which he has held for twenty-eight years, Mr. P. Jenner Verrall, M.B., B.C.Camb., F.R.C.S., has been appointed by the Council of the College to be his successor.

At a meeting of directors, held on Monday last, Dr. Dagmar F. Curjel was appointed outdoor house surgeon at the Glasgow Royal Maternity and Women's Hospital; and Dr. Sara A. Watson was appointed outdoor house surgeon at the West End branch for three months.

At a recent meeting of the Metropolitan Asylums Board the Hospitals Committee reported the appointment of the following gentlemen as assistant medical officers in the Infectious Hospitals Service, viz.: W. J. Hart, L. C. Le Blanc, J. R. C. Mackintosh, D. Porter, and I. H. Sewart.

Rates of Subscription: Twelve months, 6/6; Six months, 4/-; Three months, 2/-, post free to any address in the United Kingdom. Abroad, Twelve months, 8/8; Six months, 5/-; Three months, 2/6, post free.—Address The Subscription Dept., "The Hospital," The Hospital Building, 28/29 Southampton Street, Strand, London, W.C.

